# Enhancing the Virulence of a Fungal Entomopathogen Against the Brown Planthopper by Expressing dsRNA to Suppress Host Immune Defenses

**DOI:** 10.3390/microorganisms13112484

**Published:** 2025-10-30

**Authors:** Chenping Lan, Zhiguo Hu, Xiaoping Yu, Zhengliang Wang

**Affiliations:** 1 Zhejiang Provincial Key Laboratory of Biometrology and Inspection and Quarantine, College of Life Sciences, China Jiliang University, Hangzhou 310018, China; 2 Key Laboratory of Microbiological Metrology, Measurement and Bio-Product Quality Security (State Administration for Market Regulation), China Jiliang University, Hangzhou 310018, China

**Keywords:** *Metarhizium anisopliae*, *Nilaparvata lugens*, Spätzle, RNAi, transgenic strain

## Abstract

The use of fungal entomopathogens, such as *Metarhizium anisopliae*, is a promising alternative for pest biocontrol but suffers the disadvantage of a relatively slower killing speed when compared with chemical pesticides. *Nilaparvata lugens* (brown planthopper, BPH) is a destructive sap-sucking pest that seriously threatens rice production worldwide. In the present study, we characterized a key immune-regulating protein, Spätzle (SPZ), encoding gene *NlSPZ5* in BPH, and constructed a transgenic strain of *M. anisopliae* that expressed a specific dsRNA targeting the *NlSPZ5* gene for enhancing the fungal virulence. Expression pattern analysis revealed that *NlSPZ5* was expressed with the highest levels in the second-instar nymphs and hemolymph and could be largely activated by *M. anisopliae* infection. Microinjection of dsNlSPZ5 resulted in a markedly decreased survival rate and increased susceptibility to fungal infection in BPH. Notably, a transgenic strain of *M. anisopliae* expressing dsNlSPZ5 could effectively suppress the target gene expression and promote fungal proliferation in BPH upon fungal challenge. Compared to the wild-type strain, the transgenic fungal strain exhibited significantly enhanced insecticidal efficacy against BPH without compromising mycelial growth and sporulation. Our results demonstrate that fungal entomopathogens used as a delivery vector to express dsRNAs targeting insect immune defense-associated genes can effectively augment their virulence to the host insect, providing clues to develop novel pest management strategies through the combination of RNAi-based biotechnology and entomopathogen-based biocontrol.

## 1. Introduction

The brown planthopper (BPH) *Nilaparvata lugens* Stål is one of the most destructive pests of paddy, which damages rice plants directly by sucking the phloem sap and indirectly by transmission of diverse plant-pathogenic viruses [1]. Under favorable environmental conditions, BPH infestations can result in devastating yield and economic losses [2]. At present, the control of BPH primarily depends on chemical pesticides. However, the long-term and large-scale use of chemical pesticides causes widespread insecticidal resistance, severe environmental pollution, and human health risk [3,4]. Therefore, there is an urgent need to search for environmentally friendly alternative methods for BPH control.

The use of entomopathogenic fungi represents a safe and effective alternative strategy for controlling insect pests, especially sap-sucking hemipteran pests [5]. Nowadays, several fungal candidates, such as *Beauveria bassiana* and *Metarhizium anisopliae*, have demonstrated great potential for BPH biocontrol [6,7,8]. However, compared to chemical pesticides, fungal kill action against target pests shows a relatively slower killing speed, which severely limits the large-scale application of fungal insecticidal agents. Besides the environmental factors, the insecticidal efficacy of fungal entomopathogens can be restrained by the innate immune system of the target pest [9,10]. Accumulating studies have shown that suppressing insect immune defenses can augment the virulence of insect fungal pathogens [11,12,13]. For instance, overexpression of an immune inhibitor serpin gene, *Spn43Ac* from *Drosophila melanogaster*, in *B. bassiana* significantly enhanced the fungal virulence against the wax moth *Galleria mellonella* [14].

RNA interference (RNAi) is a post-transcriptional gene silencing mechanism that has been harnessed to develop pest control technology [15]. By introducing double-stranded RNA (dsRNA) targeting host survival-associated genes into the insect body, RNAi-mediated gene silencing can result in significant insect mortality with high specificity [16]. Exploring stable and effective dsRNA delivery methods is a key initial step in the RNAi-mediated strategy for pest control. Currently, there are three main ways of dsRNA delivery, including direct injection or feeding, genetic modification of host plants, and the use of microbial agents [17]. Of those, injection and oral feeding of dsRNA are mainly used under laboratory conditions, as dsRNA synthesized in vitro is difficult to keep stable for a long time. Transgenic plants expressing dsRNA can effectively downregulate insect target genes and lead to insect death, but this raises concerns about the safety of genetically modified (GM) crops [18]. Microbial agents, especially fungal entomopathogens, are ideal vectors for dsRNA delivery due to the additive or synergistic effects of RNAi and fungal infection [19]. Several studies have shown that transgenic fungal entomopathogen strains expressing dsRNA to silence essential insect genes display great potential for field pest control [20,21,22,23]. For example, overexpressing dsRNAs targeting host F1F0-ATPase subunit genes significantly increased the insecticidal efficacy of a locust-specific fungal pathogen, *Metarhizium acridum* [21]. Undoubtedly, studies focusing on the development and application of this combination strategy for BPH control should be strengthened.

Insects mainly rely on their innate immunity to fight against microbial pathogens [10]. The Toll signaling pathway is a prominent and evolutionarily conserved immune cascade in insects. The cytokine-like polypeptide Spätzle (SPZ) is a key protein responsible for Toll pathway activation, leading to antimicrobial peptide (AMP) production upon pathogen challenge [24]. Beyond the model insect species *D. melanogaster*, the pivotal roles of SPZ protein involved in the immune regulation have been extensively determined in diverse insect taxa, including the mosquito *Anopheles gambiae*, the tobacco hawkmoth *Manduca sexta*, the domestic silkmoth *Bombyx mori*, and the yellow mealworm *Tenebrio molitor* [25,26,27,28]. In this study, we constructed a dsRNA expression vector and transformed it into a strain of *M. anisopliae*. An SPZ-encoding gene, *NlSPZ5,* of BPH was selected as the dsRNA target. The insecticidal efficacy of the transgenic fungal strain was then evaluated in laboratory bioassays to explore the synergy between the fungal entomopathogen and RNAi in BPH control (Figure S1).

## 2. Materials and Methods

### 2.1. Fungal Strains and Insect Stock

The entomopathogenic fungal strain *Metarhizium anisopliae* ARSEF456 (designated as Ma456 herein) was inoculated on the plates of potato dextrose agar (PDA) at a regime of 25 °C and 12:12 h (light/dark photoperiod). The *Escherichia coli* DH5α strain (Invitrogen, Shanghai, China) was used for vector construction and cultured at 37 °C in Luria–Bertani (LB) medium plus 100 μg ampicillin or 50 μg kanamycin per milliliter, depending on their resistance type. *Agrobacterium tumefaciens* AGL-1 was cultured at 28 °C in YEB medium for fungal transformation.

The BPH population originally established from a paddy field collection was maintained on the rice variety Xiushui 314 under laboratory conditions (27 ± 1 °C, 70 ± 10% relative humidity, and a 14:10 h light/dark photoperiod) in the insectary greenhouse.

### 2.2. Gene Cloning and Characterization of NlSPZ5

The nucleotide sequences of *NlSPZ5* were searched from the complete genome database (GenBank accession number AOSB00000000) and transcriptome database (http://www.ncbi.nlm.nih.gov/sra, SRX023419, accessed on 10 March 2025) of BPH using the query of *Homalodisca vitripennis SPZ5* (GenBank accession number KAG8259733). Reverse transcription PCR (RT-PCR) was performed to confirm the complete cDNA sequence. Total RNA from BPH was extracted using a TaKaRa MiniBEST Universal RNA Extraction Kit (Takara, Dalian, China) according to the manufacturer’s instructions. The RNA integrity was determined by agarose gel electrophoresis, and the RNA quantity was assessed using a NanoDrop 2000 spectrophotometer (Thermo Scientific, Waltham, MA, USA). The high-quality RNA (1 μg) sample was then reverse transcribed with a TaKaRa PrimeScript^TM^ RT reagent kit with gDNA Eraser (Takara, Dalian, China). The synthesized cDNA was diluted 10-fold and used as a template for PCR cloning of *NlSPZ5* with its specific primers (Table S1). The PCR reaction was conducted in a total volume of 50 μL containing 2 μL template DNA, 5 μL 10 × PCR buffer, 1 μL dNTPs (10 mM), 1 μL of each primer (10 μM), 0.5 μL TaKaRa LA Taq (5 U/μL), and 39.5 μL sterilized ddH_2_O. The PCR program was as follows: 94 °C for 3 min, followed by 35 cycles of 94 °C for 30 s, 56 °C for 30 s, 72 °C for 1.5 min, and 72 °C for 10 min. The PCR products were checked by agarose gel electrophoresis, cloned into the PMD19-T vector (Takara, Dalian, China), and then sequenced at Sangon Inc. (Sangon, Shanghai, China). The acquired DNA sequence was translated into protein and then compared with other insect SPZ protein sequences from the NCBI database via BLASTP (https://blast.ncbi.nlm.nih.gov/Blast.cgi, accessed on 25 March 2025). The conserved domain was predicted by SMART (https://smart.embl.de/, accessed on 25 March 2025) and InterProScan (https://www.ebi.ac.uk/interpro/search/sequence/, accessed on 25 March 2025). SignalP 5.0 (https://www.cbs.dtu.dk/services/SignalP/, accessed on 25 March 2025) was used to predict the existence of signal peptides. The subcellular location was analyzed by the online server Cell-PLoc 2.0 (https://www.csbio.sjtu.edu.cn/bioinf/Cell-PLoc-2/, accessed on 25 March 2025). The phylogenetic tree was constructed using the full-length amino acid sequences of insect SPZ proteins by MEGA X. Phylogenetic relationships were determined using the neighbor-joining (NJ) method with a bootstrap test of 1000 replicates [29]. The cDNA sequence of *NlSPZ5* was deposited in GenBank with the accession number PQ373585.

### 2.3. Quantitative Real-Time PCR (qRT-PCR) Analysis of NlSPZ5

To profile the temporal transcription pattern of NlSPZ5 across the BPH development stages, the samples were separately collected from the eggs, nymphs (1st- to 5th-instar), and adults (24 h newly emerged male and female adults). For tissue-specific expression analysis, samples from different tissues, including the ovary, gut, fat body, and hemolymph, were dissected from the 24 h newly emerged female adults under a stereomicroscope. To quantify the transcription levels of *NlSPZ5* in BPH response to topical fungal infection, the 5th-instar nymphs of BPH were subjected to fungal challenge and sampled at different days post-infection (1 to 7 days). For fungal exposure, aerial conidia of Ma456 were collected from 7-day-old cultures on PDA plates and adjusted to a final concentration of 1 × 10^8^ conidia/mL in 0.02% Tween 80 solution. A cohort of 100 nymphs was sprayed with 1 mL conidial suspension following the previous protocol [7]. BPH sprayed with an equal volume of 0.02% Tween 80 served as a control. All sprayed nymphs were reared in situ in a growth chamber, and the surviving nymphs were sampled daily for 7 days from the Ma456-infected and the control group, respectively.

Total RNA was separately extracted from each developmental stage-specific, tissue-specific, and fungal-infected sample. The methods for RNA extraction and cDNA synthesis were the same as described above. The transcription levels of *NlSPZ5* and 18S rRNA (used as an internal standard) were assessed by qRT-PCR with paired primers listed in Table S1. All qRT-PCR assays were performed with SYBR^®^ Premix Ex Taq^TM^ (Takara, Dalian, China) under the following conditions: an initial denaturation for 30 s at 95 °C, followed by 40 cycles of 5 s at 95 °C and 30 s at 60 °C, and a final step for the generation of melting curves. The relative expression level of *NlSPZ5* was estimated using the 2^−ΔΔCt^ method [30]. The whole experiment was repeated three times.

### 2.4. RNAi and Bioassays

The double-stranded RNA (dsRNA) of *NlSPZ5* was synthesized in vitro according to the manufacturer’s instructions of the MEGAscript T7 transcription kit (Ambion, Austin, TX, USA). The dsRNA of the green fluorescent protein gene (dsGFP) was synthesized using the plasmid pCAMBIA1302 as a template and used as a negative control. Specific primers for the dsRNA synthesis are listed in Table S1. The specificity of dsNlSPZ5 was assessed by blasting its sequence against the entire BPH genome. The quality and quantity of the double-stranded RNA (dsRNA) products were assessed by 1% agarose gel electrophoresis and a NanoDrop 2000 spectrophotometer.

The 5th-instar nymphs of BPH were collected and anesthetized on ice. Then, 40 nL of dsRNA (5000 ng/µL) was injected into each BPH individual using a microinjector (Nanoliter 2000 Injector, WPI Inc., Sarasota, FL, USA). At least 40 BPH nymphs in each treatment group were used to assess effects on survival rates. RNAi-mediated inhibition efficiency for target gene expression was determined at 1, 3, and 5 days after dsRNA microinjection using the qRT-PCR method mentioned above. The expression level was quantified relative to the value of the nymphs injected with dsGFP. For co-treatments of RNAi and fungal infection, the 5th-instar nymphs of BPH were injected with 200 ng of dsNlSPZ5 and then topically sprayed with Ma456 conidial suspension (1 mL of 1 × 10^8^ conidia/mL) or 0.02% Tween 80 (Macklin, Shanghai, China). Control nymphs were injected with the same dose of dsGFP. BPH mortality rates after co-treatments were recorded daily for 10 days. Each experiment was conducted in triplicate.

### 2.5. Expression Vector Construction and Fungal Transformation

The plasmid p0380-bar, which carries a phosphinothricin resistance cassette, was used as a backbone to construct the dsRNA expression vector [31]. Briefly, an intron spacer sequence from the *M. anisopliae* cutinase gene, which was flanked with *Bam*HI and *Hin*dIII restriction sites, was artificially synthesized and inserted into the *Bam*HI/*Hin*dIII digested plasmid p0380-bar, generating the plasmid p0380-bar-in. Two fragments with a length of 506 bp corresponding to the sense and antisense sequences of *NlSPZ5* were separately amplified by PCR using *NlSPZ5* cDNA as the template and paired primers with proper restriction sites (Table S1). The fungal promoter P*trpC* sequence from *Aspergillus nidulans* was fused to the *NlSPZ5* sense sequence using overlap extension PCR with the primers listed in Table S1. The fusion product P*trpC*-sense was flanked with *Eco*RI at the 5′-end and *Bam*HI at the 3′-end, respectively. Similarly, the fusion fragment of the *NlSPZ5* antisense sequence and *A. nidulans* T*trpC* terminator sequence (antisense-T*trpC*) flanked with *Hin*dIII and *Kpn*I was synthesized. Subsequently, the two fusion fragments P*trpC*-sense and antisense-T*trpC* were successively introduced into the plasmid p0380-bar-in, linearized with proper restriction enzymes, respectively, yielding the final plasmid p0380-dsNlSPZ5 for *NlSPZ5* dsRNA expression.

The constructed plasmid was transformed into *A. tumefaciens* AGL-1 and then transformed into Ma456 using the method previously described [21]. Mutant colonies grown on selective plates were screened based on their *bar* resistance to phosphinothricin (200 μg/mL) and sequentially identified via PCR and RT-PCR with paired primers for three consecutive generations (Table S1). The recombinant with the best genetic stability was selected for subsequent phenotypic analyses and bioassays.

### 2.6. Phenotypic Analyses of Transgenic Fungal Strains

To assess mycelial growth rate, aliquots of 1 µL of 1 × 10^7^ conidia/mL suspension of the transgenic fungal strain and wild-type strain were spotted centrally onto PDA plates (9 cm diameter), and colony diameters were cross-measured after incubation for 14 days at 25 °C and 12:12 h (light/dark photoperiod).

To estimate conidia production, aliquots of 100 µL of 1 × 10^7^ conidia/mL suspension of each strain were evenly spread on cellophane-overlaid PDA plates and incubated for 14 days at 25 °C. Conidial yield was then estimated as the number of conidia/cm^2^ colony by washing off conidia into 1 mL of 0.02% Tween 80 from each of the colony discs (5 mm diameter) through supersonic vibration and determining conidial concentration in the suspension using microscopic counts in a hemocytometer.

To evaluate insecticidal efficacy, aliquots of 1 mL 1 × 10^8^ conidia/mL suspension of each strain were applied by topical spray against BPH 5th-instar nymphs following the protocol previously described [7]. Control insects were sprayed with an equal volume of 0.02% Tween 80. BPH mortality rates were daily monitored. The time–mortality observations were subjected to probit analysis to estimate the median lethal time (LT_50_) of each fungal spray against BPH. Each treatment was conducted in triplicate with 40 insects per replicate, and the bioassays were repeated three times.

To determine the effect of fungal *NlSPZ5*-dsRNA expression on the transcription of the target gene in BPH, the 5th-instar BPH nymphs infected by the wild-type and transgenic fungal strains were collected at 1, 2, 3, 4, and 5 days post-infection (dpi). RNA extraction, cDNA synthesis, and qRT-PCR were performed as described above.

To detect the antifungal activity of the induced hemolymph of BPH, the hemolymph samples of 5th-instar BPH nymphs (*n* = 100) infected with the wild-type and transgenic fungal strains for 4 days were separately collected. The quantitative PCR (qPCR) was conducted to determine the loads of fungal hyphal bodies in the hemolymph with the Ma456-specific primers (Table S1). The BPH 18S rDNA gene was used as an internal reference. Meanwhile, the hemolymph collected from 1 to 5 dpi was subjected to RNA extraction and cDNA synthesis, and the expression levels of downstream AMP-encoding genes were quantified by qRT-PCR analysis.

### 2.7. Statistical Analysis

Survival curves were generated using the Kaplan–Meier method, and the log-rank test was used for comparison. The data of gene expression levels, egg productions, egg hatching rates, BPH mortalities, and microbial loads from the repeated experiments were expressed as mean ± standard error. Data were statistically analyzed using DPS v7.05 software [32]. Prior to statistical analysis, the Shapiro–Wilk test or F test was performed to test the normality and homogeneity of all variances. Student’s *t*-test was used to analyze differences between two groups, while one-way analysis of variance (ANOVA) was applied to evaluate significant differences among three or more groups. Differences were considered to be significant at *p* < 0.05.

## 3. Results

### 3.1. Features of the NlSPZ5 Gene and the Deduced Amino Acid Sequence

**Figure 1:**
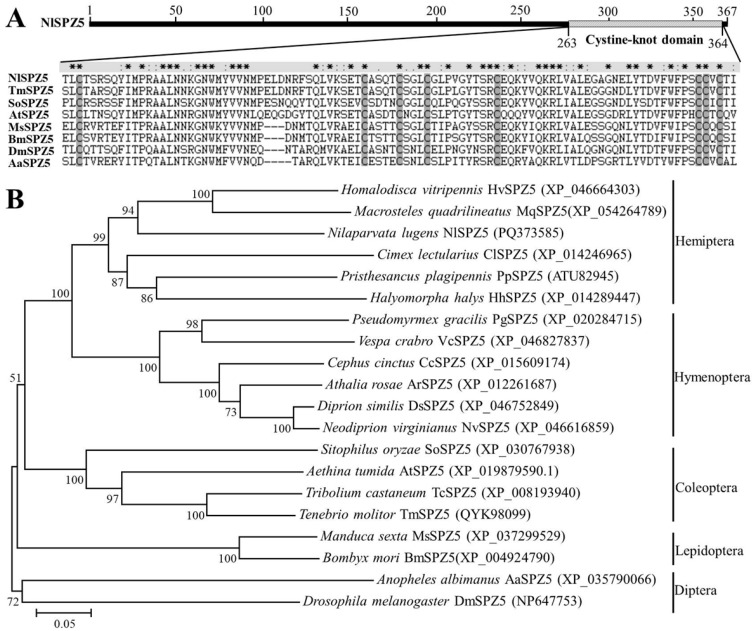
Structural and phylogenetic analysis of NlSPZ5. (**A**) Structural comparison of the cystine-knot domain of NlSPZ5 with other insect SPZ proteins. TmSPZ5: *Tenebrio molitor* SPZ5; SoSPZ5: *Sitophilus oryzae* SPZ5; AtSPZ5: *Aethina tumida* SPZ5; MsSPZ5: *Manduca sexta* SPZ5; BmSPZ5: *Bombyx mori* SPZ5; DmSPZ5: *Drosophila melanogaster* SPZ5; AaSPZ5: *Anopheles albimanus* SPZ5. The asterisk indicates invariant residues; colons or periods denote conservative replacements. (**B**) Phylogenetic comparison of NlSPZ5 with other insect SPZ proteins using the neighbor-joining method. Numbers on branches indicate bootstrap values. The GenBank accession number for each insect SPZ protein is parenthesized.

The putative coding sequence of *NlSPZ5* (GenBank accession number PQ373585) was identified by searching the *BPH* genome and transcriptomic database using the query of *H. tripennis SPZ5* and then verified via RT-PCR amplification with paired primers (Table S1). The full-length cDNA sequence of the *NlSPZ5* gene is 1104 bp, encoding 367 amino acids with the predicted molecular weight of 41.8 kDa and an isoelectric point of 8.7. Sequence comparison showed that *NlSPZ5* shares a moderate sequence identity with other insect SPZ members, such as *Homalodisca vitripennis* SPZ5 (XP_046664303, identity = 54.8%) and *Macrosteles quadrilineatus* SPZ5 (XP_054264789, identity = 53.1%). The amino acid sequence analysis revealed that the NlSPZ5 protein contains a predicted signal peptide consisting of 33 amino acid residues at the N-terminal region and a typical cystine-knot domain at the C-terminus (which binds to the Toll receptor), including eight cysteine residues that stabilize the structures via three intrachain and interchain disulfide bonds. A putative endoprotease cleavage site, Arg257, was also found at the C-terminus (Figure 1A). Subcellular localization prediction results indicated that the NlSPZ5 protein is localized in the nucleus. The phylogenetic analysis showed that NlSPZ5 clustered together with other hemipteran SPZ proteins but was quite distinct from those of lepidopterans and dipterans in the neighbor-joining tree (Figure 1B).

### 3.2. Expression Patterns of the NlSPZ5 Gene

**Figure 2:**
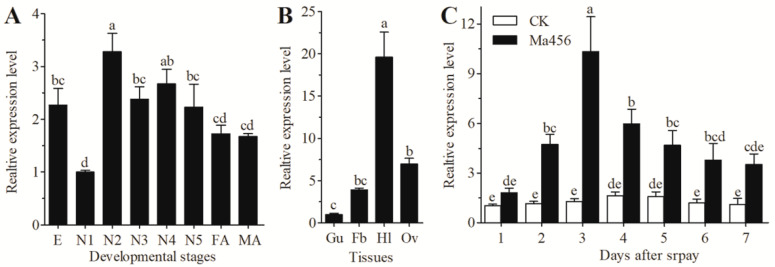
The spatiotemporal and fungal-induced expression patterns of *NlSPZ5,* determined by qRT-PCR. (**A**) Relative expression levels of *NlSPZ5* across different developmental stages. E: eggs; N1–N5: first- to fifth-instar nymphs; FA: female adults; MA: male adults. (**B**) Relative expression levels of *NlSPZ5* in different tissues. Fb: fat bodies; Gu: guts; Hl: hemolymph; Ov: ovaries. (**C**) Expression patterns of *NlSPZ5* at different time points after fungal infection. CK: spray with 0.02% Tween 80; Ma456: spray with *M. anisopliae* conidia. Different letters above bars indicate a significant difference (*p* < 0.05, one-way ANOVA test).

The expression patterns of *NlSPZ5* in different development stages, various tissues, and fungal-infected conditions are illustrated in Figure 2. As a result of qRT-PCR analysis, the expression level of *NlSPZ5* in the first-instar nymphs was significantly lower than that in the eggs and other nymphal stages. For instance, the transcription level of *NlSPZ5* in the eggs and fourth-instar nymphs was 2.3- and 2.7-fold higher than that in the first-instar nymphs, respectively. The highest expression of *NlSPZ5* level was observed in the second-instar nymphs (Figure 2A). With respect to tissue expression patterns, the *NlSPZ5* expression levels were highest in the hemolymph, followed by the fat bodies, ovaries, and guts in female adults. The mRNA expression in the hemolymph was 5.0- and 2.8-fold higher than that in the fat bodies and ovaries, respectively (Figure 2B). The expression of *NlSPZ5* in the fifth-instar BPH nymphs was significantly upregulated after topical infection with the entomopathogenic fungus Ma456 within 7 dpi. The expression level peaked at 3 dpi in response to the Ma456 challenge but tended to decrease with the extension of post-infection time (Figure 2C).

### 3.3. NlSPZ5 Gene Silencing Increased BPH Susceptibility to Fungal Infection

**Figure 3:**
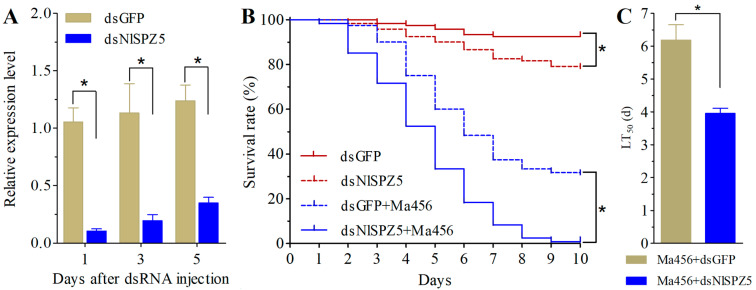
Effect of *NlSPZ5* silencing on the BPH survival and susceptibility to *M. anisopliae* infection. (**A**) RNAi efficiency of *NlSPZ5* at different time points after dsNlSPZ5 injection. The asterisk indicates a significant difference (*p* < 0.05, *t*-test). (**B**) The survival rates of BPH nymphs treated with dsRNA injection alone or combined with fungal infection during a 10-day period. The asterisk indicates a significant difference (*p* < 0.05, log-rank test). (**C**) The LT_50_ values of BPH co-treated with dsRNA injection and fungal infection. The asterisk indicates a significant difference (*p* < 0.05, *t*-test).

Microinjection of dsNlSPZ5 could significantly inhibit the expression level of *NlSPZ5* in the fifth-instar nymphs of BPH. For instance, an approximate 90% reduction and more than 70% decrease were achieved in the dsNlSPZ5-injected group compared to the dsGFP control group at 1 and 5 days post-treatment, respectively (Figure 3A). RNAi-mediated knockdown of *NlSPZ5* caused a significant decrease in the survival rate of the fifth-instar BPH nymphs. Compared with control nymphs treated with dsGFP, nymphs injected with dsNlSPZ5 exhibited a 10.8% and 15.3% decrease in the survival rate at day 7 and day 10, respectively. Additionally, the results of bioassay experiments showed that the ability to resist fungal infection in the fifth-instar nymphs of BPH was significantly decreased by *NlSPZ5* silencing. The survival rate of BPH nymphs after 7 days co-treated with dsNlSPZ5 injection and fungal infection was 8.3%, which was significantly lower than that for control insects (37.5%) (Figure 3B). The LT_50_ value for dsNlSPZ5+Ma456 co-treatment was 4.0 days. In contrast, the LT_50_ estimate was 6.2 days for dsGFP+Ma456 co-treatment (Figure 3C).

### 3.4. Transgenic Fungus Expressed dsRNA of the NlSPZ5 Gene

**Figure 4:**
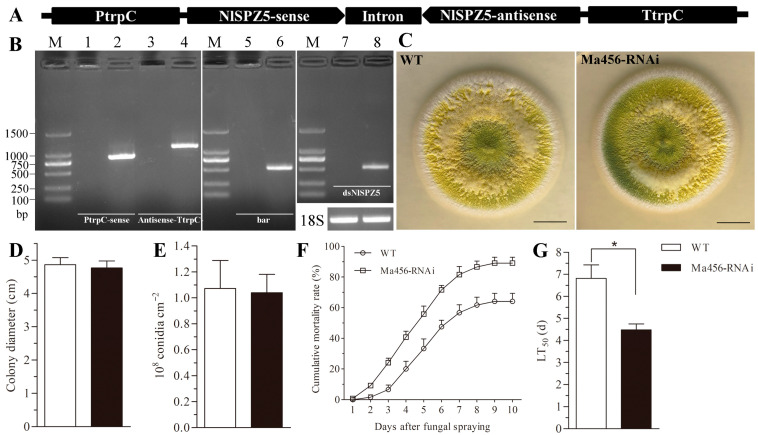
Construction of a transgenic fungal strain expressing dsNlSPZ5 and insecticidal efficacy evaluation. (**A**) Diagram for constructing a dsRNA expression vector targeting the *NlSPZ5* gene of BPH. (**B**) Identification of a transgenic fungal strain expressing dsNlSPZ5. M: DNA marker; Lanes 1–6: amplification of PtrpC-sense, antisense-TtrpC, and bar using genomic DNA of wild-type and transgenic strains, respectively; Lanes 7 and 8: amplification of dsNlSPZ5 using cDNA of wild-type and transgenic strains, respectively. 18S: 18S rRNA of *M. anisopliae*. (**C**) Fungal colony grown on a PDA plate at 25 °C for 14 days. (**D**) Colony diameters and (**E**) conidial yields measured from the PDA plates at 25 °C for 14 days. (**F**) Cumulative mortality rates of the 5th-instar nymphs of BPH after fungal infection. (**G**) LT_50_ (days) for fungal virulence of each strain against BPH nymphs. The asterisk above bars indicates a significant difference (*p* < 0.05, *t*-test).

The dsRNA expression vector p0380-dsNlSPZ5 targeting the *NlSPZ5* gene of BPH was successfully constructed, in which the sense and antisense of *NlSPZ5* sequences were separately ligated to the 5′- and 3′-end of an intron spacer, forming a hairpin RNA expression cassette (sense + intron + antisense) under the control of the constitutive fungal promoter P*trpC* (Figure 4A). The constructed vector was introduced into Ma456 using the *A. tumefaciens*-mediated transformation method. The positive recombinants were screened on selective PDA plates and verified by PCR and RT-PCR using the bar-specific, *NlSPZ5* sense- and antisense-specific primers (Table S1). As a result, a transgenic fungal strain with good genetic stability was selected and designated as Ma456-RNAi. The targeted fragments could be amplified from the genomic DNA and cDNA of the Ma456-RNAi strain, while no amplification bands were detected in the wild-type strain Ma456 (Figure 4B).

### 3.5. Biological Evaluation of the Transgenic Fungal Strain

To test whether the integration of a dsRNA expression cassette had an impact on the fungal basic characteristics, the mycelial growth and conidiation capacity were compared between the wild-type and transgenic strains. As shown in Figure 4C, the colony morphology of the wild-type and recombinant strains was similar. After 14 days of growth on PDA plates at 25 °C, both the fungal colonies achieved similar sizes with an average diameter of 4.8 cm and exhibited yellow-green color (Figure 4D). Similarly, no significant difference in the conidiation capacity was observed between the wild-type and transgenic strains. Both fungal strains exhibited the conidial yield of 1.1 × 10^8^ conidia/cm^2^ after 14 days of growth on PDA plates (Figure 4E).

### 3.6. Virulence of the Transgenic Fungal Strain Against BPH

The wild-type and transgenic strains were bioassayed for their virulence to the fifth-instar nymphs of BPH in laboratory conditions. As shown in Figure 4F, the mortality rates of BPH nymphs for the transgenic strain were significantly higher than those for the wild-type strain during a 10-day observation period. For instance, the corrected mortalities at 6 and 8 dpi were 71.7% and 86.7% for the transgenic strain, while the rates at the same post-treatment time were 47.5% and 61.7% for the wild-type strain, respectively. Probit analysis revealed the LT_50_ value for the transgenic strain was 4.5 days, which was 34.2% shorter than the estimate for the wild-type strain (6.8 days) (Figure 4G).

### 3.7. Repressed NlSPZ5 Expression in BPH Infected by Transgenic Fungal Strain

**Figure 5:**
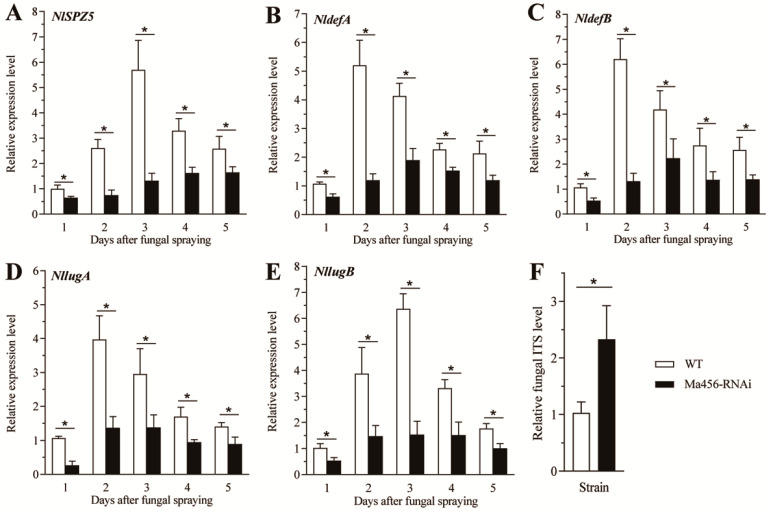
Effect of transgenic fungal strain expressing dsNlSPZ5 on target gene expressions and fungal proliferation in the hemolymph of the 5th-instar BPH nymphs. (**A**–**E**) Relative transcription levels of *NlSPZ5* and four downstream AMP genes in the hemolymph of BPH nymphs during the period of 1–5 days post-infection. (**F**) The relative load of fungal hyphal bodies in the hemolymph of fungal-infected BPH nymphs at 4 dpi. The asterisk above bars indicates a significant difference (*p* < 0.05, *t*-test).

To evaluate the silencing effect of the transgenic strain on the target gene expression, the transcription pattern of the *NlSPZ5* gene was monitored during the course of fungal infection. Compared with the wild-type strain, the transgenic strain resulted in a significantly lower *NlSPZ5* expression (Figure 5A). For instance, the transcription levels of *NlSPZ5* were repressed by 35.1–76.8% in BPH nymphs in response to the transgenic strain relative to the wild-type strain challenge over a 5-day time course, strongly indicating that the transgenic strain bearing the dsRNA expression cassette could effectively knock down the target gene expression in its host insect.

### 3.8. Reduced Antifungal Defense in BPH Infected by Transgenic Fungal Strain

The effect of the NlSPZ5-dsRNA expression in the transgenic strain on the transcription of four downstream AMP genes (*NldefA*, *NldefB*, *NllugA*, and *NllugB*) and fungal proliferation in the host hemolymph was investigated. Similarly to the expression pattern of *NlSPZ5* presented above, its downstream AMP-encoding genes were all significantly repressed after dsRNA-expressing strain infection relative to the wild-type strain (Figure 5B–E). For instance, at 2 dpi, the transcription levels of *NllugA* and *NllugB* in the hemolymph of BPH nymphs infected with the Ma456-RNAi strain were significantly decreased by 65.4% and 61.8% when compared to the control nymphs infected by the wild-type strain. Additionally, the fungal proliferation in the host hemolymph was found to be attenuated in BPH after 4 days post-infected transgenic strain. As illustrated in Figure 5F, the abundance of fungal hyphal bodies in the hemolymph of the BPH nymphs infected with the Ma456-RNAi strain was obviously increased in comparison to the Ma456-infected nymphs.

## 4. Discussion

The application of dsRNA targeting host survival-associated genes has been proven to be an effective and eco-friendly strategy of plant protection as an alternative to traditional chemical insecticides [15,16]. Biocontrol based on the use of microbial insect pathogens is another sustainable tool for pest control and is compatible with other strategies, including the RNAi-based method [13,33]. In the present study, combining the advantages of the above two strategies, we constructed a transgenic entomopathogenic fungal strain that expressed a specific dsRNA targeting the host immune defense-associated gene. The results revealed that the transgenic fungal strain effectively knocked down the target gene expression during the fungal infection and displayed a significantly enhanced insecticidal virulence against the rice pest BPH.

Under natural conditions, fungal infection of host insects begins with the adhesion of conidia to the host cuticle, followed by conidial germination and cuticle penetration for entry into the host hemocoel, where the fungus transforms as hyphal bodies to evade the host’s immune defenses and rapidly proliferates via yeast-like budding until host death [34]. Obviously, overcoming the immune defense response of the host insects is a crucial prerequisite for fungal virulence. Hence, insect immune-related genes could serve as a valuable genetic resource for the genetic improvement of the fungal insect pathogen. The Toll pathway is a critical and conserved immune defense against the invading microbial pathogen in insects, which is activated by the SPZ protein [24,35]. Our previous transcriptional data revealed a significant increase in the expression of four SPZ-encoding genes in BPH when challenged by Ma456 [7]. The qRT-PCR analysis in the present study validated that the *NlSPZ5* gene was drastically induced during the Ma456-infection course, strongly suggesting that *NlSPZ5* plays vital roles in the regulation of immune response in BPH against fungal infection. The RNAi-mediated silencing of *NlSPZ5* following fungal infection enhanced the susceptibility of BPH nymphs to Ma456 compared with the control group, indicating a synergistic effect was generated by the combination of dsRNA injection and fungal infection. These findings are in agreement with the results in the most recently published reports [12,13,36]. For instance, the mortality of the cotton boll weevil (*Anthonomus grandis*) treated with *M. anisopliae* after microinjection of dsRNA targeting host immune-related genes was significantly higher than that in the non-RNAi pre-treatment group [13].

Screening of effective RNAi target genes is a prominent step in the RNAi-mediated pest control strategy [37]. A growing body of studies has revealed that genes associated with insect immune response are important candidate RNAi targets for pest control [38,39,40,41]. For instance, RNAi silencing of two immune-related genes (*LgPGRP-LB2a* and *LgToll-5-1a*) in the soybean pod borer (*Leguminivora glycinivorella*) led to relatively low larval survival rates and abnormal development, while knocking down the genes involved in both cellular and humoral immunity in the locust (*Locusta migratoria*) caused physiological defects, ultimately resulting in insect death [39,40]. RNAi-mediated gene silencing showed that the survival rate of BPH was significantly decreased by the repression of *NlSPZ5*, suggesting that this immune-associated gene could be a potential RNAi target for BPH control. There is always a concern about the risk of off-target effects of dsRNA pesticides, which arise from sequence matches between the dsRNA and the homologous genes [15]. To mitigate this risk, the specificity of the dsNlSPZ5 sequence was assessed by blasting its sequence against the whole BPH genome, and no long stretches of nucleotide identity were detected beyond the target sequence. Nevertheless, a comprehensive RNAi biosafety evaluation under laboratory and field conditions is still necessary for further investigation.

The application of RNAi-mediated pest control relies on a stable delivery system that could ensure dsRNA is being effectively delivered into the target insect [17,18,19]. Nowadays, nanoparticle-enabled, microorganism-mediated, and plant-mediated methods have been established for dsRNA delivery [42,43,44]. Our data demonstrate that the transformation of a fungal entomopathogen to express dsRNA targeting a host immune defense-related gene can augment the fungal virulence to BPH. The expression level of the target gene *NlSPZ5* was significantly inhibited after infection by the transgenic fungal strain compared to the wild-type strain. SPZ protein is a key protein responsible for Toll pathway activation, leading to AMP production upon pathogen infection [27,45]. Expectedly, four AMP-encoding genes also had severely repressed expression when challenged by the transgenic fungal strain. These results strongly imply that the transgenic fungus could release dsRNA during the infection process, thereby weakening the BPH immune defense responses and ultimately facilitating fungal invasion. However, the delivery mechanisms of how the fungal cell releases the dsRNA into the host insects still remain unclear, warranting extensive investigation in the future.

## 5. Conclusions

In summary, the *NlSPZ5* gene was cloned and characterized in the rice pest BPH, which plays vital roles in host immune defense against fungal infection and can serve as a potential RNAi target for BPH control. The use of transgenic fungal entomopathogens as vectors expressing dsRNA targeting *NlSPZ5* can effectively weaken BPH’s immune defense and significantly augment the virulence to BPH.

## Data Availability

The original contributions presented in this study are included in the article/Supplementary Material. Further inquiries can be directed to the corresponding author.

## Supplementary Materials

The following supporting information can be downloaded at https://www.mdpi.com/article/10.3390/microorganisms13112484/s1, Figure S1: The experimental design for using a fungal pathogen as a delivery vector to express dsRNA targeting the BPH immune gene *NlSPZ5*; Table S1: Specific primer pairs used in this study.
