# Supplementary material for: Enhancing the Virulence of a Fungal Entomopathogen Against the Brown Planthopper by Expressing dsRNA to Suppress Host Immune Defenses
Source: Microorganisms. 2025 Oct 30;13(11):2484. doi: 10.3390/microorganisms13112484 (PMC12654220; doi:10.3390/microorganisms13112484)

Figure S1. The experimental design for using fungal pathogen as a delivery vector to express dsRNA targeting BPH immune gene *NlSPZ5*.

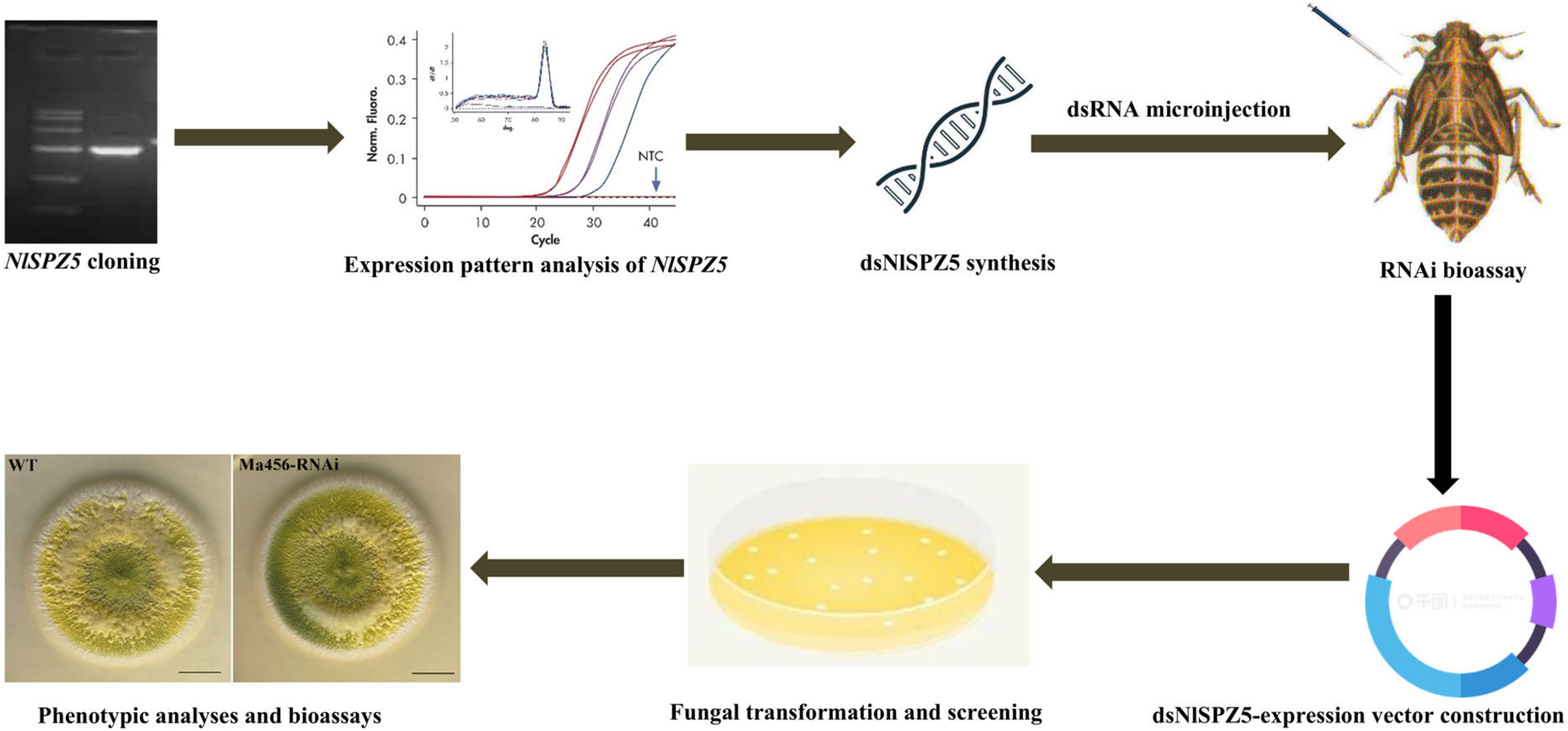

Supplement: Supplementary file 1 [file microorganisms-13-02484-s001.zip › Figure S1.pdf]
