# Supplementary material for: Enhancing the Virulence of a Fungal Entomopathogen Against the Brown Planthopper by Expressing dsRNA to Suppress Host Immune Defenses
Source: Microorganisms. 2025 Oct 30;13(11):2484. doi: 10.3390/microorganisms13112484 (PMC12654220; doi:10.3390/microorganisms13112484)
Supplement: Supplementary file 1 [file microorganisms-13-02484-s001.zip › Table S1.pdf]

Table S1. Specific primer pairs used in this study.

Primers	Sense/Antisense primer sequences (5′-3′)	Purpose
cNlSPZ5-F/R	ATGGATAGGCGCTTCTCTCTGGATC / TCATCCGTTCTGCGTGATTTGACAA	Gene cloning
qNlSPZ5-F/R	ACGACGGCGACAAGTTGAGACAG / GGTCCTGGTCGAGATTGACGACATA	qRT-PCR analysis
q18S-F/R	GTAACCCGCTGAACCTCC/ GTCCGAAGACCTCACTAAATCA	
qNldefA-F/R	GCCTCTGTGATGGCACTGTA/TGGTTCGGAGTCACCCATTG	
qNldefB -F/R	TGCCTCTGTAATGGCACTGTA/GGAGTCACCCATTTGCTGTTG	
qNllugA-F/R	TCGACCACCTTCGCTAAGCA/GGTTTCTGGTAAACTCCT	
qNllugB-F/R	GCCTATGCAAGGCTGCATCA/ GGGTTCAGCTTCGGGTGC	
dsNlSPZ5-F/R	GGATCCTAATACGACTCACTATAGGTCAACTATTCCCAGCCTCAAGTAGG /GGATCCTAATACGACTCACTATAGGGAAGACATCGGTGTAGAGTCGGTTG	dsRNA synthesis
dsGFP-F/R	GGATCCTAATACGACTCACTATAGGGATACGTGCAGGAGAGGAC/ GGATCCTAATACGACTCACTATAGGGCAGATTGTGTGGACAGG	
Intron-F/R	CGCGGATCCGTTAACCCTAACCAATTCAA/ CCCAAGCTTGGTGAGTCGTGTCAGCACAG	Vector construction
PtrpC-F/R	CCGGAATTCGTCGACAGAAGATGACATTGAAGGA/ CCTACTTGAGGCTGGGAATAGTTGAATGCTTGGGTAGAATAGGTAAGTCA	
senNlSPZ5-F/R	TGACTTACCTATTCTACCCAAGCATTCAACTATTCCCAGCCTCAAGTAGG/ CGCGGATCCGAAGACATCGGTGTAGAGTCGGTTG	
antiNlSPZ5-F/R	CCCAAGCTTGAAGACATCGGTGTAGAGTCGGTTG/ TTTGATGATTTCAGTAACGTTAAGTTCAACTATTCCCAGCCTCAAGTAGG	
TtrpC-F/R	CCTACTTGAGGCTGGGAATAGTTGAACTTAACGTTACTGAAATCATCAAA/ CGGGGTACCAAGAAGGATTACCTCTAAACAAGTG	
id-senNlSPZ5-F/R	GTCGACAGAAGATGACATTGAAGGA/ ATGCTTGAATTGGTTAGGGTTAAC	Transformant identification

id-antiNlSPZ5-F/R	CTAACCAATTCAAGCATGTGCTCG/ AAGAAGGATTACCTCTAAACAAGTG	
Bar-F/R	AGCCCAGAACGACGCCCGGCCGACA/ GAAGTCCAGCTGCCAGAAACCCACG	
id-dsNlSPZ5-F/R	TCAACTATTCCCAGCCTCAAGTAGG/ ATGCTTGAATTGGTTAGGGTTAAC	
Ma18S-F/R	GCCGTAATGATGAATAGGGACA/ CGTACTGCCATAGCAATACTGAC	
qITS-F/R	GGGGTAGCCCCTCAAGTCCCCTGC/ GGGGTTGGCTCCTGTTGCGAGTGC	qPCR analysis
